# Human Subjects Protection in the Era of the Revised Common Rule

**DOI:** 10.31486/toj.20.5011

**Published:** 2020

**Authors:** Guest Editors Joseph L. Breault, Lydia Bazzano

**Affiliations:** ^1^ Former Chair, Institutional Review Board, and Department of Family Medicine, Ochsner Clinic Foundation, New Orleans, LA; ^2^ The University of Queensland Faculty of Medicine, Ochsner Clinical School, New Orleans, LA; ^3^ Current Chair, Institutional Review Board, and Department of Internal Medicine, Ochsner Clinic Foundation, New Orleans, LA

The last several years have been a time of significant change for the clinical research enterprise, not only at the national level with the revised regulations, but also at the local level with the growth and transitions in our own health system. As the current and former executive chairs of the Ochsner Institutional Review Board (IRB) system, we thank all of the authors and peer reviewers who made this special issue possible. Because of the revised federal IRB regulations (45 CFR §46, Subpart A) that became effective in January 2019, active review is underway at all US IRBs and human research protection programs about how the new regulations should be understood, implemented, and operationalized. We hope this special edition will assist many as they work through the various issues that have been raised by these changes. Highlights of this special edition issue include the following:

White reflects on the fundamental reason we have IRBs and why human subject protection is important.

Gordon discusses the basic ethical concepts of vulnerability in research and suggests that vulnerability is not a yes/no determination but a spectrum of seriousness and consequences of situations and context.

Butcher's editorial gives a community member's perspective on IRB membership, explaining how participation gives members of the public the opportunity to ensure that the safety and interests of patients are represented.

Rosenfeld contributes two papers: an editorial promoting the use of computers as cognitive extenders for IRB decision-making, not just to automate existing processes but also to reduce unwanted variability, and a manuscript that articulates the need for ongoing quality assessment and continuous improvement in IRB decision-making. His measures of board decision quality can help us all to improve our processes.

Biggio's article on research in pregnant subjects notes that the recent Common Rule revision removed pregnant women from the vulnerable population classification, but aspects of consent requirements may still create barriers that should be reduced.

LeCompte and Young review the revised Common Rule changes to the consent process and form. Their review and example of one institution's consent form template will be helpful to many.

Gartel, Scuderi, and Servay review how research coordinators work together with the IRB to implement changes to a consent form necessitated by the revised Common Rule.

Williams and Colomb review important considerations for the IRB when acting as a privacy board to issue Health Insurance Portability and Accountability Act (HIPAA) waivers. Most exempt research may still require HIPAA waivers.

Freehan and Garcia-Diaz review investigator responsibilities in clinical research based on US Food and Drug Administration regulations. The investigator's role is not only to conduct high-quality, meaningful scientific research but also to maintain public trust and subject protection.

Bass and Maloy provide a structure for determining if a project is human subjects research, a quality improvement project, or both. Their detailed review, table of comparisons, and questions to ask to help with the decision will prove valuable to all. They also tackle understanding of broad consent, a new concept under the revised Common Rule, and the flexibility it may allow.

Breault and Knafl review pitfalls and safeguards in industry-funded research using guidance from professional standards, medical societies, and institutional policies.

Walch-Patterson takes a practical look at the revised Common Rule requirements for exempt research and limited IRB review.

Matrana and Campbell look at how IRBs can adapt their processes to adjust to precision medicine technologies based on genetic mutations. They discuss umbrella, basket, and adaptive trial designs for the evolution of a study as new data are discovered.

Finally, we remind readers that through letters to the editor, the process of clarification and the expression of differing opinions can make this edition even more useful, and we invite you to consider submitting letters about the articles presented here.

